# Prevalence and determinants of TB infection in a rural population in northeastern Myanmar

**DOI:** 10.1186/s12879-020-05646-8

**Published:** 2020-11-30

**Authors:** Theint Theint Lwin, Tawatchai Apidechkul, Jongkon Saising, Panupong Upala, Ratipark Tamornpark, Chalitar Chomchoei, Fartima Yeemard, Wipob Suttana, Rachanee Sunsern

**Affiliations:** 1 School of Health Science, Mae Fah Lunag University, Chiang Rai, 57100 Thailand; 2 Center of Excellence for Hill Tribe Health Research, Mae Fah Luang University, Chiang Rai, Thailand

**Keywords:** Tuberculosis, Tuberculin skin test, Associated factors, Myanmar, Rural population

## Abstract

**Background:**

Tuberculosis (TB) is a major human threat, as evidenced by the large numbers of cases and deaths, particularly in developing countries with poor economic and educational statuses. Myanmar has one of the highest TB burdens in the world, but no TB information is available for people living in the rural northeastern regions of Myanmar. The present study estimated the prevalence of TB and identified factors associated with TB infection in people living in rural communities in Shan State.

**Methods:**

A cross-sectional study was performed to gather information from participants. People aged 18–59 years who lived in the three areas with the highest numbers of TB cases in Shan State in northeastern Myanmar were included in the study population. A simple random method was used to select the sample from the villages. A validated questionnaire was used for data collection in face-to-face interviews after obtaining signed informed consent from the selected participants. The Mantoux tuberculin skin test (TST) was administered to detect TB infection, and a result that was 10 mm or greater after 48 h was considered positive. Chi-squared tests and logistic regression were used to identify the associations between the variables at a significance level of α = 0.05.

**Results:**

A total of 303 participants were recruited for the study; 64.7% were females, and the mean age was 37 years (SD = 12.5). Most participants were Burmese (25.4%), and 14.95% were Shan. Sixty-three participants (20.8%) had a positive TST. Four variables were associated with TB infection in the multivariate model. Males had a greater chance of TB infection than females (AOR = 2.51; 95% CI = 1.32–4.76). Participants who were ever married had a greater chance of TB infection than participants who were single (AOR = 3.93; 95% CI = 1.18–13.00). Participants who used wood and charcoal as their main sources of energy for cooking had a greater chance of TB infection than participants who used electricity (AOR = 4.23; 95% CI = 1.25–9.64). Participants who had a low level of TB prevention and care knowledge had a greater chance of TB infection than participants with a high level of TB prevention and care knowledge (AOR = 4.49; 95% CI = 1.88–10.72).

**Conclusions:**

Public health programs that focus on improving knowledge of TB prevention and care and avoiding the use of wood and charcoal as the primary sources of energy for cooking, particularly in males and ever-married individuals, are urgently needed.

## Background

Tuberculosis (TB) is a major public health burden [1, 2], particularly in developing countries [3], and it is one of the top 10 most lethal diseases worldwide. The World Health Organization (WHO) [1] estimated that 10 million (9.0–11.1 million) people suffered from TB infection in 2018, and it forecasted that more than 40 million cases will be reported globally between 2018 and 2022 [2]. Generally, TB infection occurs in major vulnerable populations, such as children with poor nutritional status [4] and people with poor immune systems, particularly people who are infected with human immunodeficiency virus (HIV) [5, 6]. The development of TB is related to other specific population characteristics, such as poverty [7], poor education [8], poor access to health care [9] and residence in cities or countries undergoing civil conflict [10]. Therefore, TB is a human health problem that also reflects inequity in human society, particularly access to public services and quality of life in different populations.

Myanmar is ranked 139th in the world economic rankings, and it is classified as a poor country (lower-middle income economy), with a gross domestic product (GDP) per capita of $6509 in 2018 [11]. There were approximately 52.6 million people from more than 135 ethnic groups living in Myanmar in 2019 [12]. Shan is a Myanmar state with a relatively large population of minority groups, who account for 10.0% of the Myanmar population and live in the northern region, especially along the border of northern Thailand [13]. These groups live in poor economic and educational conditions, and there is civil conflict between the central Myanmar government and the minority populations in the northern region of Myanmar. The WHO reported that there were 137,972 new and relapsed TB cases in 2018 in Myanmar [14]. Myanmar was ranked as one of the 20 countries with the highest TB burdens in the world with a total TB incidence of 181,000 (119,000-256,000) cases (338/100,000 population) [14], which indicates that these groups of people are obviously vulnerable to TB infection.

However, there is no public health information on TB in particular minority groups in northeastern Myanmar. Based on medical information from Thai hospitals located along the border of Thailand and Myanmar, which people in Shan State generally use when they have a medical problem, more than 150 new TB cases are diagnosed and treated annually [15]. Greater than 80.0% of TB cases are reported in people ranging from 18 to 59 years [15]. However, several aspects of the treatment process, including case management, require at least 6 months for a complete treatment cycle, and patients often must be admitted to a hospital, particularly in the early period [16]. Thailand has an excellent health care system and health insurance. However, the universal coverage scheme only covers people who are Thai citizens, and it does not cover charges for any medical treatment or care in a public hospital accessed by non-Thai citizens [17]. Therefore, all TB patients from outside of Thailand are asked to pay for medical treatment and care [18], and treatment is not affordable for patients from Myanmar. The magnitude of the TB problem and its determinants in these populations must be assessed before proper public health interventions may be implemented in these populations. The ultimate global goal of the United Nations (UN) mission is to eradicate TB by 2030 [19].

Therefore, the current study estimated the prevalence of TB infection in a community in the rural northeastern area of Myanmar and determined the factors associated with TB infection.

## Methods

### Study design

A cross-sectional design was used to collect data from the participants.

### Study setting

The study was performed in the three villages with the highest TB incidence rates in Myanmar, according to a published report [20]. These villages were in the Ta Chi Leik District, Shan State, Myanmar.

### Study population

People aged 18–59 years, which was the age group with the highest reported TB incidence rate, who lived in the study area constituted the study population [20].

### Eligible population

People aged 18–59 years who had lived in the study area for at least 3 years were eligible for the study. People who could not provide essential information on the questionnaire or who refused to undergo TB detection using a tuberculin skin test (TST) were excluded from the study.

### Sample size

The sample size was calculated using the standard formula for a cross-sectional design [21]:

$$ \mathrm{n}=\left[{{\mathrm{Z}}^2}_{\alpha /2}\mathrm{PQ}\right]/{\mathrm{e}}^2. $$

The value of *p* = 24.3% was taken from a study on the prevalence of latent tuberculosis infection and associated risk factors in China [22], and other values were q = 7.57%, e = 5.0%, and Z = 1.96. After adding a 10.0% error for the study, a total of 303 individuals were needed for the analysis.

### Research instruments

A questionnaire that was validated using the item-objective congruence (IOC) method and piloted was used for data collection. It was divided into 6 parts. First, 10 questions were used to collect general information from the participants, such as age, sex, marital status, and education. Second, 13 questions were used to collect health information and factors related to TB, such as a history of Bacillus Calmette-Guérin (BCG) vaccination, a history of TB diagnosis and treatment, and medical expenses related to treatment. Third, 16 questions were used to collect data on personal risk behaviors and living environment, such as “Do you smoke?”, “Do you drink alcohol?”, and “What kind of fuel do you use for daily cooking?” Fourth, 20 questions were used to detect knowledge and attitudes of TB prevention and care. Ten questions were asked on knowledge related to TB prevention and care. Each question in this section was scored as 0 for an incorrect answer and 1 for a correct answer. Participants who received scores of 0–5 were classified as having “poor” knowledge, participants with scores of 6–8 were classified as having “moderate” knowledge, and participants with scores of 9–10 were classified as having “good” knowledge. Another 10 questions were asked on attitudes related to TB prevention and care. Each question was rated on a five-point scale ranging from totally agree to totally disagree. Positive-attitude questions were scored as 5 (i.e., for responses of totally agree), and these scores decreased based on the increasing negativity of the attitude, with a score of 0 given for answers conveying disagreement. Negative-attitude questions were scored as 0 for responses of totally agree, with scores increasing to 5 for a response of totally disagree. There were 5 negative-attitude questions and 5 positive-attitude questions. People who scored fewer than 26 points were classified as having a poor attitude, participants who scored 26–40 were classified as having a moderate attitude, and participants who scored 41 and over were classified as having a good attitude towards TB treatment and care. Fifth, 8 questions were used for the clinical assessment and PPD tests, such as “Have you had a cough for more than two weeks in the past six months?”, “Have you experienced weight loss during the past six months?”, and “Do you have a low-grade fever in the evening?”. The last part included 3 items about the TST test and was completed by a medical doctor from one of the research teams.

The questionnaire was further developed to improve its validity and reliability before use in the field. The IOC method was used to detect its validity by three external experts in the fields of infectious disease, medical immunology, and public health. Questions with scores less than 0.50 were deleted, those with scores of 0.51–0.70 were revised, and those with scores greater than 0.70 were included in the questionnaire. A pilot was performed with 15 selected individuals who had similar characteristics to the villagers in Ta Chi Leik District, Shan State, Myanmar. The purposes the pilot study were to detect the feasibility and order of the questions in the questionnaire and the reliability of the questions on knowledge and attitude. Finally, Cronbach’s alpha of the knowledge and attitude questions was 0.77.

The Mantoux TST was used to identify TB infections in the selected participants. Tuberculin-purified protein derivative (PDD), which was produced by the Thai Red Cross (Thai Red Cross Tuberculin PPD®), was used for the TST [23], with 94.0% sensitivity and 88.0% specificity.

All selected participants were injected with 0.1 mL of tuberculin PDD in the inner surface of the forearm. The induration was measured in millimeters 48 h after the injection. An induration of 10 or more millimeters was considered positive for TB infection, according to the recommendation of the Centers for Disease Control and Prevention (CDC) in the United States [24].

### Data collection procedures

The head of the Department of National TB Control of Myanmar was contacted to obtain approval for conducting research in Myanmar. Afterward, Ta Chi Leik District health officers were contacted regarding the study and were asked to communicate with 3 village headmen. The list of people who met the criteria was obtained from the village headmen: 201 people from village No. 1; 335 people from village No. 2; and 139 people from village No. 3. A random method was used to select the 303 required participants from the lists of villagers. All selected participants were provided with all essential information and were invited to participate in the study in Burmese. The questionnaire took 20 min to complete.

### Statistical analysis

Information from the questionnaires was converted into code and double entered into an Excel sheet. Analyses were performed using SPSS version 24, 2016 (SPSS, Chicago, IL). Descriptive statistics were used to describe the participants’ characteristics. Logistic regression was used to determine associations between the variables in univariate and multivariate models at the level of alpha = 0.05. The “ENTER” method was used in univariate and multivariate analyses. All independent variables in the univariate model were associated with TB infection in an independent process at the significance level of 0.05, and crude odds ratios (COR) were generated. All independent variables were included in the multivariate model simultaneously, and variables that were not significant were deleted individually, starting with the variable with the largest *p*-value (over 0.05), until only the significant variables (p-value less than 0.05) remained in the model. The model was preliminarily interpreted. Age, religion, education and ethnicity were controlled as confounding factors in the model. The final interpretations of the adjusted odds ratios (AORs) were made. During the analysis, some variables, such as attitude related to TB prevention and care and knowledge related to TB prevention and care, were grouped into smaller categories because a few samples were counted into that cell. The reason was to improve the power of the test and meet the criteria of the statistics used.

## Results

A total of 303 participants participated from three different villages: 97 participants were from village No. 1; 140 participants were from village No. 2; and 66 participants were from village No. 3. More than half of the participants were females (64.7%), married (65.7%) and Christian (56.1%). The mean age was 37.0 years, and 40.3% of the participants were Shan and Burmese. Most participants had a low education level (41.6% were not educated) and poor economic status (41.9%), with incomes equal to or less than 100,000 kyat per month ($65) (Table 1).

**Table 1:** General characteristics of the participants

Characteristics	n	%
**Total**	**303**	**100.0**
**Sex**		
Male	107	35.3
Female	196	64.7
**Age** (years)		
18–24	61	20.1
24–34	82	27.1
35–44	61	20.1
45–54	66	21.8
≥ 55	33	10.9
Mean = 37.0, SD =12.5		
**Religion**		
Buddhist	120	39.6
Christian	170	56.1
Other	13	4.3
**Ethnicity**		
Burmese	77	25.4
Shan	45	14.9
Ahka	81	26.7
Lahu	89	29.4
Other	11	3.6
**Marital status**		
Single	79	26.1
Married	199	65.7
Ever married	25	8.2
**Education**		
Illiterate	126	41.6
Primary school	24	7.9
Secondary school	33	10.9
High school	37	12.2
University	83	27.4
**Occupation**		
Unemployed	66	21.8
Unskilled labor	44	14.5
Private employer	20	6.6
Government staff	81	26.7
Own business	92	30.4
**Family income** (kyat)		
≤ 100,000	127	41.9
100,001-200,000	116	38.3
200,001-400,000	44	14.5
> 400,000	16	5.3
**No. of family members** (persons)		
≤ 5	203	67.0
6–10	92	30.4
> 10	8	2.6
**Length of residence in this village** (years)		
≤ 10	136	44.9
11–20	59	19.5
> 20	108	35.6

Fifty-one (16.8%) participants were defined as underweight. Only 56.1% had a history of receiving the BCG vaccination, 38.3% had at least one family member who had been infected with TB in the past, and most had received treatment and care at a government hospital. A few people had been screened for TB (2.0%), and one-fourth had been tested for HIV/AIDS. Most of the participants lived less than 5 km from a health care center (97.0%) but needed transportation to a health care center that cost less than 5000 kyat ($3.39) per visit. Half of the participants (48.2%) had no right to access free-of-charge medical services, and a large proportion (75.2%) experienced language barriers while attending a clinic. More than half (58.1%) of the participants had a low level of knowledge and a negative attitude (63.4%) toward TB prevention and care.

The overall prevalence of TB infection was 20.8%: 20.6% in village No. 1, 20.7% in village No. 2, and 21.2% in village No. 3 (Table 2).

**Table 2:** Medical-related information of the participants

Medical information	n	%
**BMI** (kg/m^2^)		
Underweight (< 18.5)	51	16.8
Normal (18.5–24.9)	173	57.1
Overweight (25–29.9)	64	21.1
Obese (≥30.0)	15	5.0
**History of having BCG vaccine**		
No	133	43.9
Yes	170	56.1
**Family member(s) with history regarding TB disease**		
Yes	116	38.3
No	187	61.7
**Place of TB diagnosis for those who had a history of TB disease**		
Government hospital	112	96.6
Private hospital	2	1.7
Others	2	1.7
**History of receiving TB treatment**		
Yes	113	97.4
No	3	2.6
**Completed treatment**		
Yes	111	98.2
No	2	1.8
**Length of completed TB treatment** (months)		
6	102	91.9
8	9	8.1
**Contact with someone with prolong coughing**		
Yes	140	46.2
No	163	53.8
**Place of contact history**		
Work-place	17	12.2
Camp	44	31.4
Household	79	56.4
**History of TST test**		
Yes	6	2.0
No	287	94.7
Do not know	10	3.3
**HIV/AIDS test**		
Yes	84	27.7
No	219	72.3
**Diabetes mellitus**		
Yes	12	4.0
No	291	96.0
**Distance to the health center** (miles)		
≤ 5	294	97.0
> 5	9	3.0
**Transportation cost** (kyat)		
< 5000	177	58.4
5000-10,000	114	37.6
> 10,000	12	4.0
**Language barrier to accessing health care services**		
Yes	75	24.8
No	228	75.2
**Getting free-of-charge medical costs**		
Yes	157	51.8
No	146	48.2
**Knowledge related to TB prevention and care**		
Low	176	58.1
High	127	41.9
**Attitude related to TB prevention and care**		
Negative	192	63.4
Positive	111	36.6
**Tuberculin skin test results after 48 h**		
Negative (< 10 mm)	240	79.2
Positive (≥ 10 mm)	63	20.8
*Village No.1*	*20/97*	*20.6*
*Village No.2*	*29/140*	*20.7*
*Village No.3*	*14/66*	*21.2*

The health behaviors of the participants revealed that 22.4% smoked, 13.9% consumed alcohol, 3.3% used heroin, and 5.9% used amphetamines (Table 3).

**Table 3:** Health behaviors

Health behaviors	n	%
**Smoking**		
Yes	41	13.5
Quit	33	10.9
No	229	75.6
**Number of cigarettes per day** (piece)		
≤ 10	49	66.2
> 10	25	33.8
**Alcohol use**		
Yes	42	13.9
No	261	86.1
**Frequency of alcohol use**		
Nearly daily	3	7.0
Weekly	17	39.5
Monthly	23	53.5
**Heroin use**		
Yes	10	3.3
No	293	96.7
**Amphetamine use**		
Yes	18	5.9
No	285	94.1
**Opium use**		
Yes	8	2.6
No	295	97.4

More than half of the participants (59.4%) lived in brick houses. A total of 77.6% cooked indoors, and 73.6% used wood and charcoal as their main sources of fuel for indoor cooking (Table 4).

**Table 4:** Environmental factors of the participants

Factors	n	%
**Type of housing**		
Bamboo and thatch house	42	13.9
Wooden house	81	26.7
Brick house	180	59.4
**Main source of fuel for cooking**		
Electricity	66	21.8
Gas	14	4.6
Wood and charcoal	223	73.6
**Indoor cooking**		
No	68	22.4
Yes	235	77.6
**Type of fuel for indoor cooking**		
Electric	80	26.4
Wood and charcoal	223	73.6

The univariate model found that 12 variables were associated with TB infection: sex, marital status, education, having family member(s) with a history of TB disease, smoking, number of cigarettes smoked per day, heroin use, amphetamine use, main source of energy for cooking, distance to health center, experiencing language barriers when accessing health care, and knowledge of TB prevention and care (Table 5).

**Table 5:** Univariate and multivariate analyses to detect factors associated with TB infection

Characteristics	Yes		No		OR	95% CI	***p***-value	AOR	95% CI	***p***-value
	n	%	n	%						
**Age** (years)										
18–24	10	16.4	51	83.6	0.45	0.16–1.23	0.121			
24–34	14	17.1	68	82.9	0.47	0.18–1.21	0.119			
35–44	14	23.0	47	77.0	0.68	0.26–1.77	0.436			
45–54	15	22.7	51	77.3	0.67	0.26–1.73	0.415			
≥ 55	10	30.3	23	69.7	1.00					
**Sex**										
Male	32	29.9	75	70.1	2.27	1.29–3.99	0.004^*^	2.51	1.32–4.76	0.005*
Female	31	15.8	165	84.2	1.00			1.00		
**BMI** (kg/m^2^)										
Underweight (< 18.5)	11	21.6	40	78.4	0.75	0.20–2.84	0.679			
Normal (18.5–24.9)	33	19.1	140	80.9	0.64	0.19–2.16	0.481			
Overweight (25–29.9)	15	23.4	49	76.6	0.84	0.23–3.03	0.792			
Obese (≥30.0)	4	26.7	11	73.3	1.00					
**Religion**										
Buddhist	19	15.8	101	84.2	0.30	0.08–1.02	0.054			
Christian	39	22.9	131	77.1	0.48	0.14–1.54	0.215			
Other	5	38.5	8	61.5	1.00					
**Ethnicity**										
Burmese	11	14.3	66	85.7	1.66	0.19–14.34	0.642			
Shan	8	17.8	37	82.2	2.16	0.24–19.37	0.491			
Ahka	23	28.4	58	71.6	3.96	0.48–32.76	0.201			
Lahu	20	22.5	69	77.5	2.89	0.35–24.02	0.324			
Other	1	9.1	10	90.9	1.00					
**Marital status**										
Single	10	12.7	69	87.3	1.00			1.00		
Married	45	22.6	154	77.4	2.01	0.96–4.23	0.064	1.82	0.77–4.29	0.167
Ever married	8	32.0	17	68.0	3.24	1.11–9.47	0.031^*^	3.93	1.18–13.00	0.025*
**Education**										
Illiterate	34	27.0	92	73.0	2.41	1.14–5.10	0.020^*^			
Primary school	6	25.0	18	75.0	2.18	0.71–6.69	0.172			
Secondary school	5	15.1	28	84.9	1.16	0.37–3.66	0.789			
High school	7	18.9	30	81.1	1.52	0.54–4.31	0.424			
University	11	13.3	72	86.7	1.00					
**Occupation**										
Unemployed	12	18.2	54	81.8	1.00					
Unskilled labor	9	20.4	35	79.6	1.15	0.44–3.03	0.766			
Private employer	1	5.0	19	95.0	0.23	0.02–1.94	0.180			
Government staff	14	17.3	67	82.7	0.94	0.40–2.20	0.887			
Own business	27	29.4	65	70.6	1.86	0.86–4.03	0.111			
**Family income** (kyat)										
≤ 100,000	33	26.0	94	74.0	1.05	0.31–3.49	0.932			
100,001-200,000	21	18.1	95	81.9	0.66	0.19–2.26	0.512			
200,001-400,000	5	11.4	39	88.6	0.46	0.10–2.04	0.313			
> 400,000	4	25.0	12	75.0	1.00					
**Number of family members** (persons)										
≤ 5	40	19.7	163	80.3	1.71	0.20–14.36	0.618			
6–10	22	23.9	70	76.1	2.20	0.25–18.38	0.472			
> 10	1	12.5	7	87.5	1.00					
**History of BCG vaccination**										
No	29	21.8	104	78.2	1.12	0.64–1.95	0.701			
Yes	34	20.0	136	80.0	1.00					
**Family member(s) with history of TB disease**										
Yes	34	29.3	82	70.7	2.25	1.28–3.96	0.005^*^			
No	29	15.5	158	84.5	1.00					
**HIV/AIDS testing**										
Yes	17	20.2	67	79.8	0.95	0.51–1.78	0.883			
No	46	21.0	173	79.0	1.00					
**Diabetes mellitus**										
Yes	3	25.0	9	75.0	1.28	0.33–4.88	0.715			
No	60	20.6	231	79.4	1.00					
**Smoking**										
Yes	14	34.2	27	65.8	2.30	1.11–4.77	0.024^*^			
Quit	7	21.2	26	78.8	1.19	0.48–2.94	0.693			
No	42	18.3	187	81.7	1.00					
**Number of cigarettes smoked per day** (piece)										
0–10	10	20.4	39	79.6	1.00					
> 10	11	44.0	14	56.0	3.06	1.07–8.77	0.037^*^			
**Alcohol use**										
Yes	8	19.0	34	81.0	0.88	0.38–2.01	0.764			
No	55	21.1	206	78.9	1.00					
**Heroin use**										
Yes	5	50.0	5	50.0	4.05	1.13–14.46	0.031^*^			
No	58	19.8	235	80.2	1.00					
**Amphetamine use**										
Yes	8	44.4	10	55.6	3.34	1.26–8.87	0.015^*^			
No	55	19.3	230	80.7	1.00					
**Opium use**										
Yes	4	50.0	4	50.0	4.00	0.97–16.46	0.055			
No	59	20.0	236	80.0	1.00					
**Main source of energy for cooking**										
Electric	4	6.1	62	93.9	1.00					
Gas	1	7.1	13	92.9	1.19	0.12–11.55	0.879	0.73	0.07–10.82	0.900
Wood and charcoal	58	26.0	165	74.0	3.15	1.89–15.63	0.001*	4.23	1.25–9.64	0.025*
**Indoor cooking practice**										
No	12	17.6	56	82.4	1.00					
Yes	51	21.7	184	78.3	1.29	0.64–2.59	0.469			
**Distance to health center** (miles)										
≤ 5	57	19.4	237	80.6	1.00					
> 5	6	66.7	3	33.3	8.31	2.01–34.25	0.003^*^			
**Language barrier during accessing health care services**										
Yes	23	30.7	52	69.3	2.07	1.14–3.78	0.016^*^			
No	40	17.5	188	82.5	1.00					
**Knowledge on TB prevention and care**										
Low	51	27.1	137	72.9	3.19	1.62–6.30	0.001^*^	4.49	1.88–10.72	0.001*
High	12	10.4	103	89.6	1.00			1.00		
**Attitude on TB prevention and care**										
Negative	48	22.2	168	77.8	1.37	0.72–2.60	0.335			
Positive	15	17.2	72	82.8	1.00					

*Significant level at α = 0.05

However, only 4 variables remained associated with TB infection in the multivariate model after controlling for age, religion, education and ethnicity: sex, marital status, main source of energy for cooking, and knowledge of TB prevention and care. Males had a greater chance of TB infection than females (AOR = 2.51; 95% CI = 1.32–4.76). Participants who were ever married had a greater chance of TB infection than participants who were single (AOR = 3.93; 95% CI = 1.18–13.00). Participants who used wood and charcoal as their main sources of energy for cooking had a greater chance of TB infection than participants who used electricity (AOR = 4.23; 95% CI = 1.25–9.64). Participants who had a low level of knowledge of TB prevention and care had a greater chance of TB infection than participants who had a high level of knowledge of TB prevention and care (AOR = 4.49; 95% CI = 1.88–10.72) (Table 5).

## Discussion

People in rural areas in Myanmar, particularly people aged 18–59 years, were living with a high prevalence of TB infection (28.0%). This population generally had a poor economic status and low education level. A large proportion of the people had low knowledge of and negative attitudes towards TB prevention and care. The coverage of BCG vaccination and TB screening was also low. Male sex, ever-married status, use of wood and charcoal as the main sources of energy for cooking, and poor knowledge of TB prevention and care were strongly associated with TB infection.

The prevalence of TB in the present study was higher than the overall TB case prevalence in Myanmar, which is 0.3%, as reported in the 2018 TB country profile from the WHO [25]. However, this result may not be completely comparable because there were different sources of data, different target populations, and different definitions. Our study estimated the prevalence of TB infection, and the WHO reported the prevalence of TB cases. The WHO reported the prevalence rates for the overall country and all age categories, and we selected villages with the highest caseloads based on data from local hospitals and focused only on people aged 18–59 years. The villages were specifically chosen because they were in the areas with the highest numbers of TB cases. The report from the WHO was based on walk-in cases in all health care centers, and the current study investigated communities based on nonclinical signs and presenting symptoms of TB infection. However, both sources of information are crucial for policy makers in Myanmar. The WHO data represent the situation nationwide, particularly the burden on health care services, and the community-based data in the present study reflected the magnitude of undetected TB cases and clearly presented the determinants of TB infection in rural Myanmar, especially ethnic minority populations. A mass screening program to identify latent TB cases in communities and providing proper treatment are recommended. Health policy makers should be concerned with strengthening health care systems, particularly improvements in infrastructure and health professional skills in TB treatment and care. Health education on TB prevention and control should be implemented for people living in remote areas.

Our study identified the factors associated with TB infection in people living in rural Myanmar. Males had a greater chance of TB infection than females. This result is supported by a 2018 systematic review on TB burden in several low- and middle-income countries, which showed that males had a greater chance of TB infection than females [26]. A study in Brazil also reported that males had a higher rate of TB infection than females in people who had a history of close contact with TB patients [27]. A prospective cohort study in Taiwan clearly showed that males had a greater chance of TB infection than females [28]. Most males had more harsh working conditions than females, and males were more likely to abuse substances. Therefore, males have more opportunity for TB exposure.

We also found that people who were divorced had a greater chance of TB infection than people who were single, which is consistent with a study in Ethiopia that showed that unmarried people were at higher risk of TB infection than married people [29]. However, a study in South Africa reported that people who were single had a greater chance for TB infection than other groups [30]. This difference may be due to the difference in the study populations. Our study investigated members of the general population living in a village, and the study in South Africa was performed with HIV/ADS patients.

One interesting result from the present study was the association of TB with the use of charcoal and wood as the main source of fuel for cooking, particularly among participants who cooked indoors. This finding is supported by a study in Ghana that reported that people who were charcoal producers and cooked indoors with charcoal were at a higher risk for TB infection than their counterparts [31]. A study in Nepal also clearly demonstrated an association between indoor cooking with charcoal and wood and later TB infection [32]. A study in India reported that people who cooked indoors were at a greater risk of TB infection than people who did not [33]. The findings from studies in Europe [34] and South Africa [35] also demonstrated that indoor cooking was a risk factor for TB, particularly people who used solid fuel (i.e., coal and biomass). People who used charcoal and wood for cooking inside houses with poor ventilation, such as the people in the villages investigated in the present study, were much more likely to contract TB.

Many studies [36, 37] reported that household contact with TB patients was a major risk factor for TB infection in family members. However, our study found that contact with family members who were diagnosed with TB infection was not a risk factor in the multivariate analysis. This difference may be because most people in these villages have a similar degree of exposure to people with TB as a result of living an area with a high prevalence of TB.

Finally, we found that people who had a poor knowledge of TB prevention and care were more at risk for TB infection than people who had a high level of knowledge. Several studies [38–41] demonstrated the effect of low knowledge of TB prevention and care on TB infection in different communities in various countries. People with less knowledge are likely to lack the skills and practices needed to prevent TB infection, unlike people who have good level of knowledge. Adequate knowledge is commonly associated with having a relatively high level of education, and people who have a high level of education are relatively more likely to engage in the practices needed to prevent TB infection. People with higher levels of education are also likely to live in a better environment because they are have a higher socioeconomic status.

The present study has a few limitations. We used the TST to identify TB infection due to the limited tools available for TB screening in the area. Even the sputum method was very difficult to manage due to the lack of medical laboratories in the study area. However, the PPD used in the study had a high sensitivity of 94.0% but a low specificity of 88.0%, which indicates the possibility of false positives. Therefore, it was recommended that people who had a positive result undergo further investigation and treatment at a local hospital if needed. There are several ethnic groups living in the study area, some of whom are not fluent in Burmese. We asked the local public health staff who were fluent in Burmese and the local language to translate on some occasions during the study.

## Conclusion

People in some communities in Shan State near the Thailand border, are living with a high prevalence of TB, particularly people aged 18–59 years. Many people use charcoal and wood as their main source of fuel for indoor cooking, and many lack adequate knowledge of TB prevention and care. Many are vulnerable to TB infection, particularly males and divorced individuals. Public health interventions that focus on mass screening using a proper tool in rural areas should be implemented to identify latent TB infection and proper treatment in communities. Collaborations between national and international organizations are urgently needed to reduce the TB burden and help meet the UN’s goal of eradicating TB in the near future.

## Data Availability

The datasets used and/or analysed during the current study are available from the corresponding author on reasonable request.

## Abbreviations

AOR: Adjusted odds ratio
AIDS: Acquired immunodeficiency syndrome
BCG: Bacillus Calmette–Guérin
CDC: Centers for Disease Control and Prevention
CI: Confidence interval
COR: Crude odds ratio
GDP: Gross domestic product
HIV: Human immunodeficiency virus
IOC: Item-objective congruence
OR: Odds ratio
AOR: Adjusted odds ratio
TB: Tuberculosis
PPD: Purified protein derivative
SD: Standard deviation
TST: Tuberculin skin test
UN: United Nations
WHO: World Health Organization
